# Genomic profiling for copy number changes in plasma of ovarian cancer patients – a new era for cancer diagnostics?

**DOI:** 10.1186/s12916-016-0741-0

**Published:** 2016-11-17

**Authors:** Vathany Kulasingam, Eleftherios P. Diamandis

**Affiliations:** 1 Department of Clinical Biochemistry, University Health Network, Toronto, ON Canada; 2 Department of Laboratory Medicine and Pathobiology, University of Toronto, Toronto, Ontario Canada; 3 Department of Pathology and Laboratory Medicine, Mount Sinai Hospital, Toronto, Ontario Canada

**Keywords:** Tumor markers, NIPT, Ovarian cancer, Non-invasive

## Abstract

A blood test that can detect human malignancy with high clinical sensitivity and specificity is highly desirable. To achieve this, a tumor marker is needed that correlates with tumor burden and that can be measured with high analytical sensitivity and specificity. Over the past decades, a number of different types of tumor markers have emerged, including proteins such as enzymes, glycoproteins, and oncofetal antigens. Besides proteins, genetic abnormalities such as mutations, amplifications, and circulating tumor DNA have served as tumor markers. Despite the diversity of such biomarkers, their acceptance and implementation into routine clinical practice requires that their use results in improvements in patient outcome. Current tumor markers used in the clinic have limited utility. As such, innovative approaches to identifying tumor markers are highly desirable and one such approach may be to look for sub-chromosomal changes in the blood of patients with ovarian cancer, as is routinely performed in prenatal screening.

Please see related article: http://bmcmedicine.biomedcentral.com/articles/10.1186/s12916-016-0667-6

## Background

Ovarian cancer is highly lethal. Current efforts to combat this disease include improved therapies and early diagnosis. Although it is known that detection of early disease can lead to prolonged survival, and even cures, most ovarian cancers are diagnosed at an advanced stage, when surgery and chemotherapy are not very effective [1]. Subsequently, new methods for early diagnosis of this cancer are highly desirable.

In an article recently published in *BMC Medicine*, Cohen and colleagues propose a new technique for early ovarian cancer diagnosis, using a routine non-invasive prenatal testing (NIPT) platform [2]. Before we analyze this paper in some detail, we summarize below some prerequisites that should be taken into account when considering ovarian cancer screening.

Ovarian cancer screening is considered for women 50–75 years of age. In this age group, the prevalence of the disease is approximately 1 in 200 women [3]. It has previously been suggested that a screening method for ovarian cancer should have a positive predictive value of at least 10 % to be viable. Assuming that a test for ovarian cancer screening has a sensitivity of 90 %, thus detecting most early ovarian cancers, a specificity of 90 % will yield a positive predictive value of around 4 %, and a specificity of 95 % will yield a positive predictive value of 8 %. Consequently, the relatively low prevalence of ovarian cancer in the screening population dictates that very high specificity (i.e., > 95 %) is required to yield a viable positive predictive value.

In the largest randomized ovarian cancer screening trial in the UK (UK Collaborative Trial of Ovarian Cancer Screening, UKCTOCS), three groups of women were included: one was based on annual multimodal screening with serum CA125 interpreted using the risk of ovarian cancer algorithm and transvaginal ultrasound (multimodal screening; MMS), the second group was based on annual transvaginal ultrasound alone (ultrasound screening; USS) and the third group received no screening. The study was powered to detect a mortality reduction of 30 %. After 11 years of follow-up, the risk of dying from ovarian cancer was slightly reduced in the MSS screening arm (by about 15–20 %) [3]. These data suggest that the reduction of mortality from ovarian cancer with the available screening methodologies is modest. Thus, the issue of screening for ovarian cancer is still controversial.

In addition, a number of investigators have examined the utility of novel serum protein biomarkers for early diagnosis, including the Early Detection Research Network (EDRN), an initiative of the National Cancer Institute. EDRN evaluated 49 candidate biomarkers for their suitability for early ovarian cancer detection in blood [4]. These data have shown that none of these serum tests or combinations have the necessary sensitivity and specificity to be used as screening tools.

More recently, it has been speculated that cell-free DNA could be an effective tumor marker that is characterized by high specificity [5]. We have recently explored the use of circulating tumor DNA for cancer diagnostics [6]. In short, it appears that circulating tumor DNA is a promising new biomarker for disease monitoring and for the selection of personalized therapy. For the most demanding application of cancer screening, this methodology could be problematic for the following reasons:

1. At early disease stages, the presence of tumor DNA in the circulation is questionable. Even if very small amounts exist, sampling on small volumes (such as 10 mL of blood) may not lead to tumor DNA retrieval, and the test will be falsely negative.
2. It is technically difficult to detect tumor DNA alternations (mutations, copy number variations (CNVs), etc.) in the presence of large amounts of non-tumor DNA, and specific techniques are required [5]. These techniques usually necessitate knowledge of the mutations to be monitored, which are usually found by sequencing tumor tissue. Without such knowledge, the depth of sequencing must be (unattainably) deep to detect the molecular changes.

## Sub-chromosomal changes: novel cancer biomarker concept

Cohen et al. used a commercially available NIPT method that identifies fetal aneuploidies by sequencing cell-free DNA in the maternal plasma. This technology is highly effective in prenatal screening for detecting fetal aneuploidies of chromosomes 21, 18, 13, X, and Y. This technique is successful because the amount of fetal DNA in the maternal circulation is rather high (e.g., around 5 % of total DNA). Other authors have previously shown that the use of NIPT can, in some cases, detect maternal malignancies at asymptomatic stages [7, 8]. Using NIPT sequencing data, sub-chromosomal changes (genomic gains or losses greater than 15 megabases (MB)) were considered by Cohen et al. as positive indicators of malignancy. In most cases they were able to map these changes to copy number variations (CNVs) reported by The Cancer Genome Atlas Research Network for ovarian cancer [9]. The NIPT sequence data were also interpreted using the routine NIPT pipeline for fetal aneuploidies.

Cohen et al. report that the routine NIPT interpretative algorithm for fetal aneuploidies was not sensitive in detecting cancers. However, the open source algorithm used to identify genomic gains or losses greater than 15 MB had 40 % sensitivity for identifying ovarian cancer, with approximately the same frequency for early (6/16) and advanced (7/16) ovarian carcinomas. At this 40 % sensitivity, the specificity was 94 % (2/32). The 12 out of 13 sub-chromosomal changes identified by this technology represented recurrent changes reported previously for high-grade serous ovarian carcinomas [9]. The authors conclude that this technology may have some utility for ovarian cancer screening or diagnosis. By refining their bioinformatic algorithm and using a larger dataset, this approach may lead to increased sensitivity.

A central question to this approach is how much tumor DNA is present in the plasma of non-pregnant populations in comparison to non-tumor DNA. If tumor DNA is much less abundant than non-tumor DNA, then this method is unlikely to be highly effective in detecting CNVs specific to tumor DNA. Unfortunately, the identified CNVs in circulating plasma DNA could not be confirmed by analysis of paired tumor DNA, since the latter was not available for testing. Consequently, we cannot say with confidence that these CNVs are indeed somatic genomic changes in tumor DNA, but their mapping to CNVs reported by The Cancer Genome Atlas is suggestive and the data look promising.

The finding of the authors that early-stage and late-stage ovarian carcinomas are detected with about the same frequency (40 %) is rather surprising because other classical tumor markers and circulating tumor DNA correlate with tumor burden (and disease stage). Usually, late-stage disease is diagnosed more effectively than early-stage disease. The authors provide potential explanations for this paradox but this finding needs to be investigated further.

Besides the sensitivity of this method, the specificity needs to be carefully examined. The authors state that technical issues with archived plasma specimens or the reference chromosome set may have led to the false positive results (2/32) in the control cohorts. Future validation studies by this group should carefully examine the pre-analytical steps in this approach to evaluate such technical issues and ensure that a standardized and robust method is in place before analysis of clinical specimens.

## Conclusions

Cohen et al. report a proof-of-concept method describing early diagnosis of ovarian cancer using sub-chromosomal changes in plasma DNA, identified with a routine NIPT platform. It is unclear if these alterations are present in tumor-derived circulating DNA. The unavailability of tumor tissue meant that the central hypothesis could not be confirmed or refuted. If clinical and analytical performance is validated in future studies, specific clinical niche needs within ovarian cancer patient management may be fulfilled with this novel approach. Using an existing clinical platform in a novel way – that is, using a platform for prenatal testing in ovarian cancer diagnosis or for monitoring therapeutic response – is attractive to both clinicians and hospital administrators. The cost savings of such an approach would be significant. But a well-designed validation study needs to be performed to both reduce pre-analytical bias and examine paired tumor tissue and plasma DNA. Such an approach may also hold promise for other cancer types and this should be examined further.

## References

[1] Ren F, Shen J, Shi H, Hornicek FJ, Kan Q, Duan Z (2016). Novel mechanisms and approaches to overcome multidrug resistance in the treatment of ovarian cancer. Biochim Biophys Acta.

[2] Cohen PA, Flowers N, Tong S, Hannah N, Pertile MD, Hui L (2016). Abnormal plasma DNA profiles in early ovarian cancer using a non-invasive prenatal testing platform: implications for cancer screening. BMC Med.

[3] Jacobs IJ, Menon U, Ryan A, Gentry-Maharaj A, Burnell M, Kalsi JK (2016). Ovarian cancer screening and mortality in the UK Collaborative Trial of Ovarian Cancer Screening (UKCTOCS): a randomised controlled trial. Lancet.

[4] Cramer DW, Bast RC, Berg CD, Diamandis EP, Godwin AK (2011). Ovarian cancer biomarker performance in prostate, lung, colorectal, and ovarian cancer screening trial specimens. Cancer Prev Res (Phila).

[5] Velculescu V, Bender E (2015). Q&A: Victor Velculescu. Out for blood. Nature.

[6] Leung F, Kulasingam V, Diamandis EP, Hoon DS, Kinzler K, Pantel K, Alix-Panabières C (2016). Circulating tumor DNA as a cancer biomarker: fact or fiction?. Clin Chem.

[7] Bianchi DW, Chudova D, Sehnert AJ, Bhatt S, Murray K, Prosen TL (2015). Noninvasive prenatal testing and incidental detection of occult maternal malignancies. JAMA.

[8] Amant F, Verheecke M, Wlodarska I, Dehaspe L, Brady P, Brison N (2015). Presymptomatic identification of cancers in pregnant women during noninvasive prenatal testing. JAMA Oncol.

[9] Cancer Genome Atlas Research Network (2011). Integrated genomic analyses of ovarian carcinoma. Nature.

